# Splenic Rupture Due to Metastasis of Breast Cancer: A Report of a Rare Case and Focused Literature Review

**DOI:** 10.7759/cureus.103422

**Published:** 2026-02-11

**Authors:** Kayra Cangoz, Muhammed Bahadir Avci, Isil Karabulut, Firathan Sarialtin, Hakan Atas

**Affiliations:** 1 Department of General Surgery, Akdagmadeni Sehit Sinan Babacan State Hospital, Yozgat, TUR; 2 Department of General Surgery, Kahramankazan State Hospital, Ankara, TUR; 3 Department of General Surgery, Kackar State Hospital, Rize, TUR; 4 Department of Radiology, Ankara Bilkent City Hospital, Ankara, TUR; 5 Department of General Surgery, Ankara Bilkent City Hospital, Ankara, TUR

**Keywords:** breast cancer, capsular rupture, emergency surgery, metastasis, splenic metastasis

## Abstract

The most common primary tumors that metastasize to the spleen include melanoma, lung, colorectal, ovarian, and breast cancers (BC). Nearly all patients with splenic metastases (SM) present with disseminated malignant disease rather than isolated involvement. We report the case of a 58-year-old female patient with BC who developed SM and subsequently died following splenic capsular rupture. The patient initially presented with biopsy-proven BC and underwent neoadjuvant therapy followed by right modified radical mastectomy. She later received adjuvant systemic therapy, radiotherapy, and tamoxifen. During follow-up, cervical lymph node metastasis was detected, and breast magnetic resonance imaging (MRI) revealed a new lesion in the contralateral breast, metastatic left axillary lymph nodes, and multiple pulmonary nodules. She subsequently underwent a left modified radical mastectomy. A few days after this second surgery, she presented to the emergency department with acute symptoms. Imaging demonstrated a hepatic metastasis, a SM, and active splenic bleeding. Emergency splenectomy was performed; however, the patient died during postoperative observation. Although SM from BC is exceedingly rare, clinicians should remain alert, particularly in patients exhibiting aggressive tumor biology or unfavorable histological subtypes. Splenic involvement may progress rapidly and can lead to life-threatening complications, including capsular rupture.

## Introduction

The spleen is the second-largest organ of the reticuloendothelial system. Several unique anatomical and immunological factors, such as the sharp angle between the splenic artery and the celiac axis, rhythmic contractions of the sinusoidal structure, the inhibitory effect of the splenic microenvironment, the absence of afferent lymphatic vessels within the splenic parenchyma, and the presence of a firm capsule, are believed to contribute to the rarity of splenic metastasis (SM) [1-4]. In a large autopsy series examining more than 2000 deaths due to solid tumors, SMs were identified in only 3% of cases, and merely 0.3%-0.7% of these were attributed to breast cancer (BC) [5].

The most common primary tumors that metastasize to the spleen include melanoma, lung, colorectal, ovarian, and BCs [6,7]. Nearly all patients with SMs have disseminated malignant disease rather than isolated involvement [6-8]. SM may present with nonspecific symptoms such as malaise, weight loss, heartburn, splenomegaly, hypersplenism, or left upper quadrant abdominal pain [4]. Splenectomy is considered the best therapeutic option and the potential curative strategy in cases of isolated SM lesions [1]. Here, we present the case of a 58-year-old female patient with BC who developed SM and subsequently died following splenic capsular rupture.

## Case presentation

A 58-year-old female patient presented to our hospital after receiving neoadjuvant therapy for biopsy-proven BC. A preoperative positron emission tomography (PET-CT) scan demonstrated a right breast lesion consistent with primary malignancy, accompanied by multiple metastatic lymph nodes (LN) in the right axilla. She subsequently underwent a right modified radical mastectomy. Histopathology examination revealed invasive breast carcinoma of no special type with focal micropapillary features. The tumor showed estrogen receptor (ER) and progesterone receptor (PR) positivity, demonstrated human epidermal growth factor receptor 2 (HER2) immunohistochemistry score of 1+, and had a Ki-67 proliferation index of 30%.

**Figure 1:**
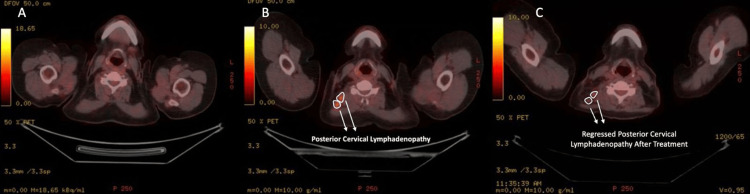
Serial axial 18F-FDG PET/CT findings demonstrating the development and regression of cervical lymph node metastasis A) Axial preoperative 18F-FDG PET/CT imaging showing no cervical lymphadenopathy B) Axial 18F-FDG PET/CT imaging performed 11 months postoperatively, demonstrating metastatic cervical lymphadenopathy (SUV value: 4.69) C) Axial 18F-FDG PET/CT imaging obtained 17 months postoperatively, after treatment, showing lymph nodes with dimensional and metabolic regression (SUV value: 2.44) 18F-FDG PET/CT: 18F-fluorodeoxyglucose positron emission tomography/computed tomography; SUV: standardized uptake value

She then received adjuvant systemic therapy, radiotherapy, and hormonal therapy with tamoxifen. At the postoperative seventh month, mammography and breast ultrasonography (US) of the contralateral breast showed normal findings. However, at the postoperative 11th month, neck US revealed LNs with malignant features and millimetric necrotic areas, the largest measuring 12 x 11 mm in the right posterior cervical chain, which had not been present on her preoperative PET-CT. Biopsy of these LNs confirmed metastatic carcinoma. Subsequent thoracoabdominal computed tomography (CT) imaging demonstrated enlarged left axillary LNs measuring 36 x 27 mm, along with smaller prepectoral LNs. A follow-up PET-CT scan showed hypermetabolic LNs in the left axillary, subpectoral, interpectoral, anterior mediastinal regions, and the posterior cervical chain, all consistent with metastases. On the PET-CT performed 17 months postoperatively, the right posterior cervical LN demonstrated a slight decrease in size following treatment (Figure 1).

**Figure 2:**
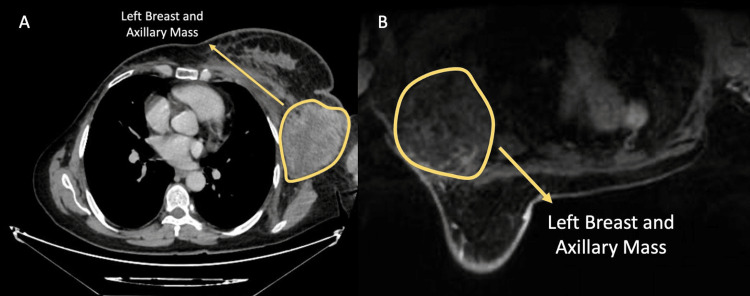
Post-contrast CT and MRI images demonstrating a left axillary mass A) Post-contrast axial CT image demonstrating a left axillary mass B) Post-contrast axial T1-weighted breast MRI image demonstrating a left axillary mass

Two years after her initial operation, control imaging revealed conglomerated metastatic LNs in the left axilla and metastatic pulmonary nodules, with no further findings on thoracoabdominal CT. Biopsy of the axillary LNs confirmed metastatic epithelial carcinoma. Two months later, a breast magnetic resonance imaging (MRI) demonstrated a 20 x 7 mm lesion in the outer quadrant of the left breast, metastatic left axillary LNs, and three nodules in the right lung measuring up to 15 mm (Figure 2). She subsequently underwent a left modified radical mastectomy, and histopathological examination revealed micropapillary carcinoma, ER-negative, PR 10%, and a Ki-67 index of 90%, along with conglomerated metastatic LNs.

**Figure 3:**
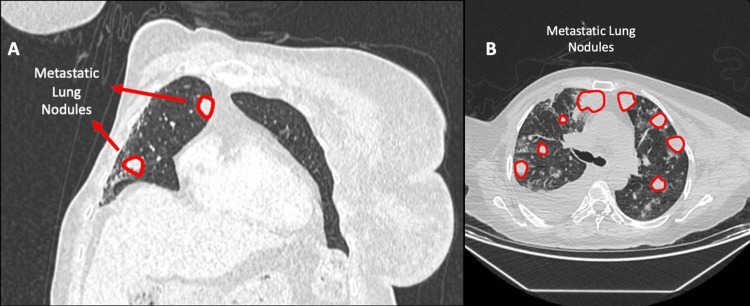
Post-contrast CT images demonstrating the progression of pulmonary metastases A) Post-contrast coronal CT image showing metastatic nodules in the right lung B) Post-contrast axial CT image obtained three months later demonstrating multiple metastatic nodules in both lungs

**Figure 4:**
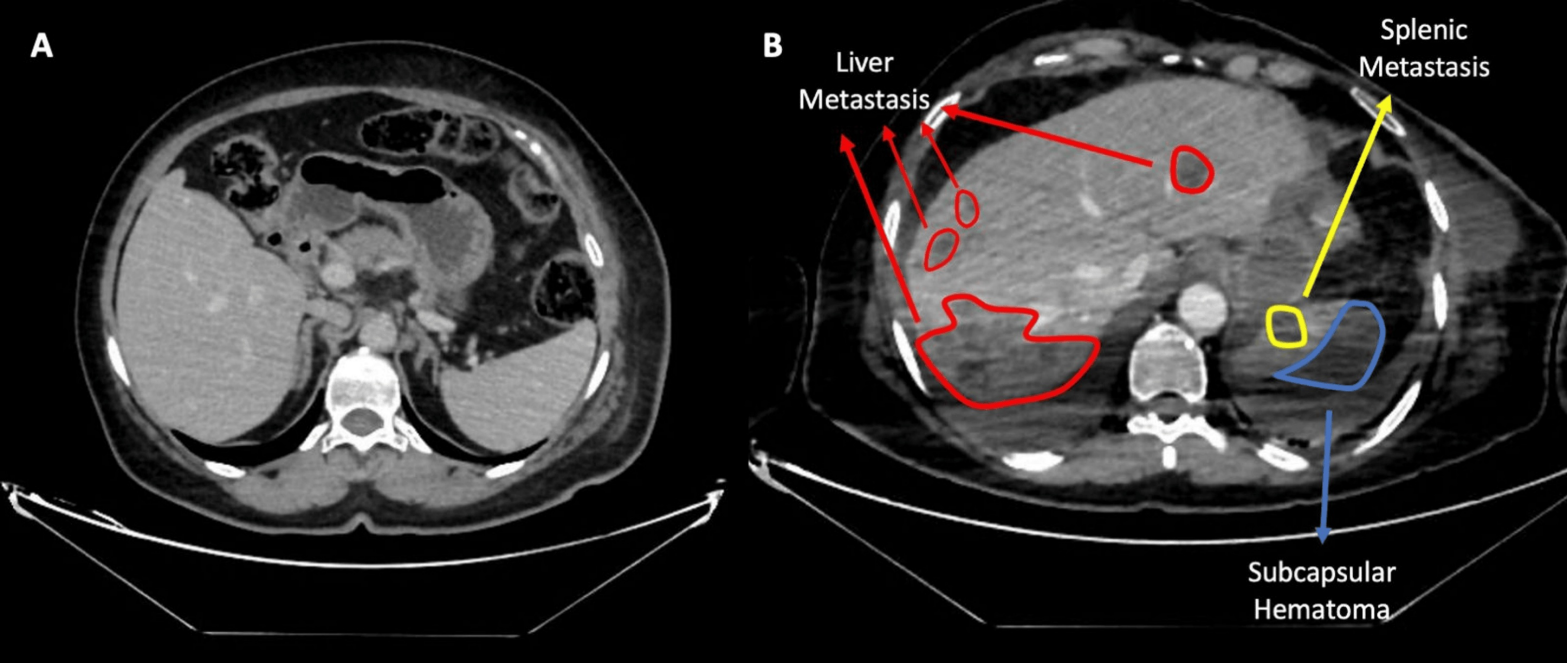
Serial post-contrast CT images demonstrating interval development of hepatic and splenic metastases with associated subcapsular hematoma A) Post-contrast axial CT image showing no metastatic lesions in the liver or spleen B) Post-contrast axial image obtained three months later demonstrating metastatic lesions in the liver and spleen, accompanied by the development of a subcapsular hematoma

**Figure 5:**
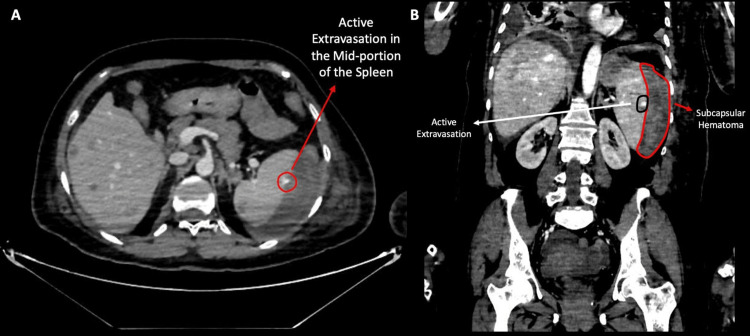
Post-contrast CT images demonstrating active splenic contrast extravasation with associated subcapsular hematoma A) Post-contrast axial CT image demonstrating a hyperdense area consistent with active splenic extravasation B) Post-contrast coronal CT image demonstrating a hyperdense area indicating active splenic extravasation together with a subcapsular hematoma

Nineteen days after her second surgery, she presented to the emergency department with dyspnea and chest pain. She was hypotensive, with a hemoglobin level of 5.3 g/dl. Subsequent CT imaging revealed a 24-mm metastatic lesion in segment VIII of the liver, a 22-mm metastatic lesion in the upper pole of the spleen, an increased number and size of pulmonary nodules, and active bleeding from the mid-portion of the spleen (Figures 3-5).

**Figure 6:**
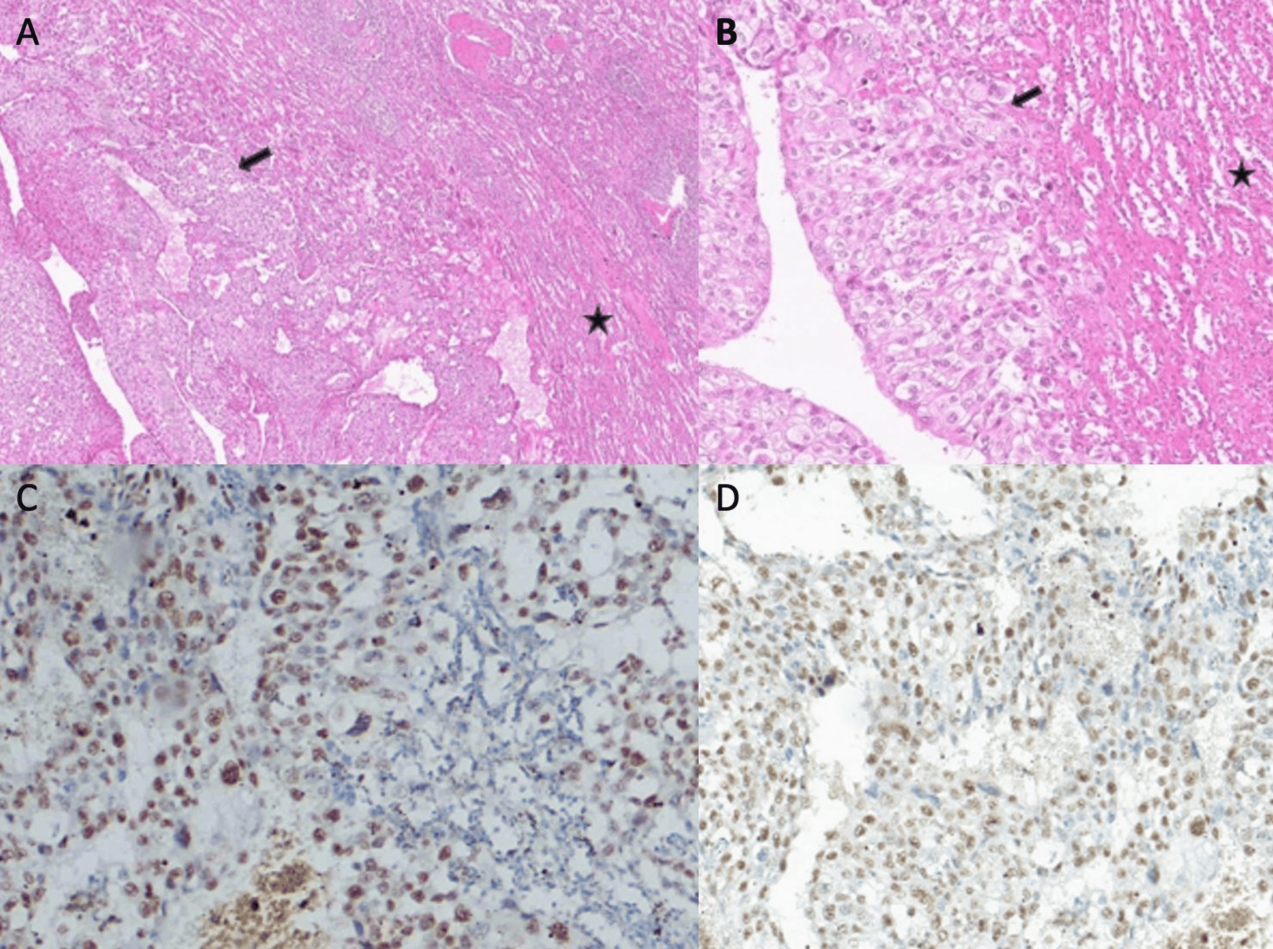
Histopathological and immunohistochemical features of splenic metastasis from breast carcinoma A) Metastatic infiltration of the splenic parenchyma by breast carcinoma (H&E, ×40; asterisk: non-neoplastic splenic tissue; arrow: metastatic tumor) B) Epithelial tumor cells arranged in solid sheets, exhibiting abundant cytoplasm and marked nuclear pleomorphism (H&E, ×200) C) Tumor cells showing positivity for estrogen receptor (ER) on immunohistochemical staining (IHC, ×200) D) Nuclear immunoreactivity for GATA3 observed within the tumor cells (IHC, ×200) H&E: hematoxylin and eosin; IHC: immunohistochemistry

The scan also demonstrated 15 mm of free perisplenic fluid and 11 cm of free fluid in the pelvis. She underwent an emergency splenectomy; however, despite surgical intervention, she died under postoperative observation. Histopathologic evaluation of the spleen revealed findings consistent with metastatic involvement (Figure 6). 

## Discussion

The incidence of SM from BC is extremely low; however, improvements in cancer care, imaging modalities, and long-term follow-up have contributed to more frequent detection in recent years, with nearly all affected patients presenting with disseminated disease [6,9]. Several studies have reported the incidence of metastatic splenic lesions. Berge identified SM in 7.1% of 7,165 autopsy examinations of patients who died from various malignancies [10]. Kraus et al. reported that, among 1,280 splenectomy specimens over a 10-year period, only 17 cases demonstrated metastatic involvement [11]. Ishida et al. found that of 24,761 patients examined by US over a seven-year period, only 0.15% had SM, none of which originated from BC, and 86.1% of these patients had disseminated disease [12]. These ratios highlight the rarity of SM and underscore the value of presenting such a case in the literature.

Xie et al. reported that among 1,009 patients with metastatic BC, only 18 (1.7%) developed SM [2]. Of these patients, 15 had died by the time of analysis, and the median age at diagnosis was 51 years, with most cases representing invasive ductal carcinoma [2]. In contrast, our patient was older than the median age described by Xie et al., and her presentation differed from the observation by Berge, who reported that younger patients tend to exhibit more aggressive primary tumors and SMs [2,10]. Li et al. further demonstrated that micropapillary carcinomas represent an aggressive phenotype, with higher rates of vascular and lymphatic invasion, LN metastasis, and poorer prognosis compared with non-micropapillary tumors [13]. In our case, the presence of micropapillary carcinoma (an uncommon subtype associated with aggressive biological behavior) and its absence in the large cohort analyzed by Xie et al. further underscore the unusual biological profile of this patient’s disease [2].

Furthermore, Xie et al. noted that 14 (77.7%) of their patients with SM were hormone receptor-positive, and that 13 (72.2%) had a Ki-67 index greater than 30% [2]. They also reported that HER2-low or HER2-negative tumors were more likely to develop splenic involvement [2]. Although such characteristics do not establish causality, our patient’s initial presentation with hormone receptor-positive, HER2 low disease and a Ki-67 index of 30% reflects a biological profile similar to that observed in patients who developed SM in the Xie et al. cohort [2]. Although a Ki-67 index of 30% has been reported as a threshold associated with a more favorable prognosis and lower tumor malignancy in BC [14], the presence of an invasive micropapillary component and nodal burden may partly explain the aggressive clinical behavior observed in this patient.

Corso et al., in their study of 15,168 patients, emphasized that contralateral breast tumors are usually considered de novo lesions because their histopathological and biological features frequently differ from those of the primary tumor [15]. They further reported that while adjuvant hormonochemotherapy and radiotherapy reduce the risk of contralateral BC, factors such as LN involvement and older age significantly increase the risk [15]. Despite receiving tamoxifen, adjuvant chemotherapy, and radiotherapy (therapies generally considered protective), our patient developed a biologically distinct contralateral tumor. When comparing the two lesions, the second tumor clearly demonstrated a more aggressive phenotype, progressing from focal micropapillary features to overt micropapillary carcinoma, from hormone receptor-positive to hormone receptor-negative status, and from a Ki-67 index of 30% to 90%, indicating a marked increase in proliferative activity consistent with an aggressive biological phenotype.

Yoshioka et al. reported that isolated SM from BC is extremely rare, with only 15 documented cases in the literature [5]. Xie et al. similarly observed no isolated SM in their cohort; all patients had disseminated disease, most commonly with synchronous liver involvement [2]. They also noted a median interval of 25.4 months between the diagnosis of BC and the detection of SM [2]. Our case parallels these findings, as the patient developed SM approximately 24 months after the initial operation and exhibited concomitant liver metastasis. Although Xie et al. suggested that SM itself does not constitute an emergency, our patient experienced a fatal outcome due to splenic capsular rupture, which developed within a short interval based on serial imaging studies, a phenomenon more commonly described in hematological malignancies but exceptionally rare in solid tumors such as BC [2].

## Conclusions

This case highlights that although SM from BC is exceedingly rare, clinicians should remain vigilant, particularly in patients exhibiting aggressive tumor biology such as high Ki-67 indices or unfavorable histological subtypes. Splenic involvement can progress rapidly and may lead to life-threatening complications, including capsular rupture. To the best of our knowledge, this is the first reported case demonstrating a rapid progression to SM followed by a fatal capsular rupture in the context of BC.
